# The Behavior of the Maize B Chromosome and Centromere

**DOI:** 10.3390/genes9100476

**Published:** 2018-10-01

**Authors:** Handong Su, Yalin Liu, Yang Liu, James A. Birchler, Fangpu Han

**Affiliations:** 1 State Key Laboratory of Plant Cell and Chromosome Engineering, Institute of Genetics and Developmental Biology, Chinese Academy of Sciences, Beijing 100101, China; shdong@genetics.ac.cn (H.S.); ylliu@genetics.ac.cn (Y.L.); yangliu@genetics.ac.cn (Y.L.); 2 University of Chinese Academy of Sciences, Beijing 100049, China; 3 Division of Biological Sciences, University of Missouri, Columbia, MO 65211, USA

**Keywords:** maize B chromosome, centromere, inactivation, reactivation, de novo centromere formation, epigenetics

## Abstract

The maize B chromosome is a non-essential chromosome with an accumulation mechanism. The dispensable nature of the B chromosome facilitates many types of genetic studies in maize. Maize lines with B chromosomes have been widely used in studies of centromere functions. Here, we discuss the maize B chromosome alongside the latest progress of B centromere activities, including centromere misdivision, inactivation, reactivation, and de novo centromere formation. The meiotic features of the B centromere, related to mini-chromosomes and the control of the size of the maize centromere, are also discussed.

## 1. Introduction

**Figure 1:**
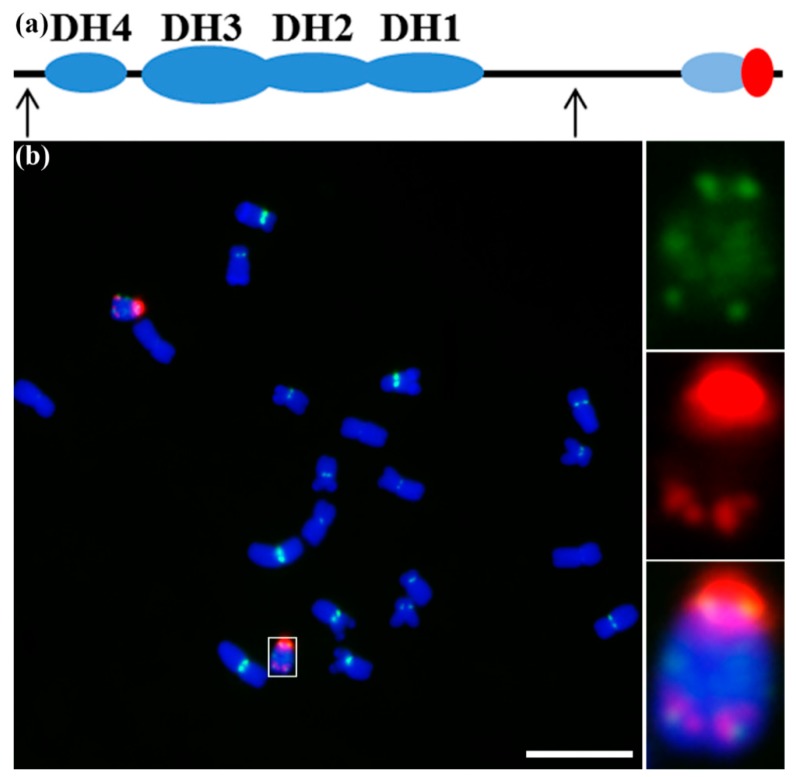
The structure and organization of the maize B chromosome and its centromere. (**a**) Diagram of the maize B chromosome with centric heterochromatin, four heterochromatic blocks (DH1–DH4) in the long arm, and two euchromatic regions—one proximal and one distal. The red oval indicates the centric heterochromatin. The light-blue oval indicates the knob signals. The two arrows indicate the two potential regions that are essential for nondisjunction. (**b**) The distribution of CentC (green) and maize B centromere repeat sequence (B-repeat) (red) signals along the maize B chromosome. The white box indicates the B chromosome, and the insets show a higher magnification view of the B chromosome. 4′,6-diamidino-2-phenylindole (DAPI)-stained chromosomes are blue. Bar = 10 μm.

The B chromosome is a non-essential chromosome that does not pair with the normal (A) chromosomes during meiosis. B chromosomes are widespread in fungi, plants and animals species [1]. The maize B chromosome is the first B chromosome to be observed [2]. Subsequent cytological observations in the pachytene stage of meiosis [3,4,5] defined the chromosome structure as including a centromeric chromatin, four heterochromatic blocks (DH1–DH4) in the long arm, and two euchromatic regions that are proximal and distal (diagram of the maize B chromosome in Figure 1a). The maize B chromosome has been studied for several decades for cytology, genetics and sequence [6]. Maize lines containing B chromosomes have been constructed and are widely used in the studies of maize genetics, engineered mini-chromosomes, gene dosage, and centromere functions [7,8]. However, the key issues on the origin, evolution and molecular mechanism of its accumulation in maize populations were still largely unknown. In this review, we will focus on the recent progress of studies on the maize B chromosome, with new insight into B centromere activities, including centromere misdivision, inactivation, reactivation, and de novo centromere formation.

## 2. The Structure and Organization of the Maize B Chromosome

The maize B chromosome is not detrimental to plant development unless it is present at approximately 15 copies and more [9]. The study of the maize B chromosome’s sequence organization is fundamental in order to understand the origin and evolution of the B chromosome. All known retrotransposon DNA sequences from maize A chromosomes were found to be distributed on the B chromosome [10]. A collection of repeat DNA sequences from A centromeres including CentC, centromere retrotransposon sequence CRM, and CentA were also found to be widely distributed on the B chromosome and, interestingly, not only concentrated in the B centromere [11]. However, several maize B chromosomal specific DNA sequences were isolated. The first one was the maize B centromere repeat sequence (B-repeats or ZmBs) with a unit size over 1.0 kb [12]. The B-repeat has similarity with the Cent4 sequence, the pericentromeric sequence on maize chromosome 4 [13]. Recent work, using de novo assembly from RNA sequencing (RNA-seq) data sets of maize B73 and B chromosomes, also identified two B-chromosome-specific long terminal repeat (LTR) retrotransposons with only partial sequences homologous to the A genome [14]. The transcriptome analysis revealed the transcription of B chromosome genes, including one of the two B-chromosome-specific LTRs. This analysis also showed that the transcription from A chromosome genes was affected by the presence of the B chromosome, as an increase of B chromosome numbers correlated with an increase of the effect [14], indicating that the B chromosome is not generally inert. The transcription of the B chromosome may occur in a condition-specific manner, like the nondisjunction property discussed below. Many sequence variations, including single nucleotide polymorphisms (SNPs) and insertions/deletions (Indels), were accumulated in the B-located gene fragments, compared with their A chromosome counterparts [14].

## 3. The Composition of the Maize B Centromere and Its Misdivision

The centromere mediates the assembly of the kinetochore, which is essential for faithful chromosome segregation during cell division [15]. Centromere identity, in most species, is determined by the presence of the histone H3 variant CENH3 [16,17]. The epigenetic mechanism for centromere specification also includes the phosphorylation of histone H2AThr133 in plants [18]. The DNA components of maize A chromosome centromeres mainly consist of two types of DNA sequences, as do most species, being the 156-bp tandem repeat CentC and CRM [8,19]. The isolated B-specific repeat sequences were confirmed to be scattered throughout, and around, the B centromere, and numerous copies had high variation. CentC and CRM sequences were embedded within the multimegabases of B-repeat in the B centromere (Figure 1b). The colocalization of the sequences with CENH3 labeling indicated they confer for centromere function, but only small portions of B-repeats were occupied by CENH3 [11,20]. In addition, the B-repeat, CentC, and CRM sequences were also located in many other distinct regions of the B chromosome (Figure 1b). Both of these results indicate that the DNA sequence alone cannot determine centromere function. What distinguishes the binding, or not, with CENH3 for the same DNA sequence is still unknown.

**Figure 2:**
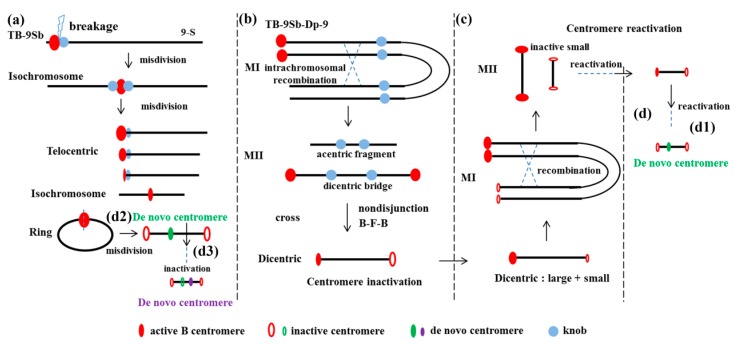
Diagrams of centromere behavior associated with the B chromosome. (**a**) The first misdivision process of a TB-9Sb chromosome produced a pseudoisochromosome; and different types of B chromosome variants formed, including telocentric chromosomes, ring chromosomes, and isochromosomes. (**b**) The TB-9Sb-Dp9 chromosome underwent intrachromosomal recombination during meiosis I, and an acentric fragment and a dicentric bridge formed during meiosis II. Centromere inactivation of the dicentric chromosome occurred from the nondisjunction, breakage-fusion-bridge (B-F-B) cycle, and the misdivision process. (**c**) A large-small dicentric chromosome underwent intrachromosomal recombination. Centromere reactivation occurred on the previously inactive small sister chromatids. (**d**) De novo centromere formation occurred in the ectopic genomic region from the reactivation of a dicentric chromosome (d1) or TB-9Sb centromere misdivision (d2) process. Inactivation occurred at the original active de novo centromere position, and a subsequent de novo formation was formed in another chromosomal region (d3).

Misdivision is the process of improper division of a univalent chromosome centromere during meiosis, resulting in isochromosome, telocentric and/or ring chromosomes [21]. A B–A translocation line TB-9Sb that involved a B chromosome and the short arm of chromosome 9 with several phenotypic markers was extensively used [22,23]. Misdivisions of the centromere of this translocation chromosome [24,25] produced a series of B centromere variant lines, which is a good model for the study of centromere function (Figure 2a). A series of telocentric chromosomes with different sized B centromeres were used to study the B centromere. The mapping of the B centromere, using Fiber-fluorescent in situ hybridization (FISH) with five B centromere misdivision derivatives, showed a 700 kb core centromere region with B-repeat, CentC, and CRM retrotransposon [20]. The transposon display with CRM2, using these misdivision lines, also generated a map of the B centromere core with 33 markers that directly interacted with CENH3. These efforts to accumulate the complete B centromere map will provide an opportunity to precisely record the process of centromere misdivision events, and to further understand the molecular basis of centromere function.

## 4. B Chromosome Nondisjunction

The accumulation mechanism of the B chromosome works to maintain it in populations. It consists of nondisjunction at the second pollen mitosis followed by preferential fertilization of the egg by the B chromosome that contains sperm [26,27]. Genetic studies have proven that the B centromere adjacent heterochromatin, and at least two other regions that are located in the very distal tip and proximal euchromatin, are essential for this process [4,6,28,29], as nondisjunction does not occur in the centromere when these regions are removed. The target site for nondisjunction may be located in B-repeat sequences, as CentC and CRM repeats are also located in A centromeres but nondisjunction does not occur for A chromosomes [5]. When an inactive B centromere was crossed with a whole B chromosome, the translocated chromosome, with the inactive B centromere attached to the short arm of chromosome 9, regained the property of nondisjunction, indicating that centromere function and B chromosome nondisjunction are two independent processes [30]. These two sites may generate transcripts or protein factors that act in trans on the B-repeat for the nondisjunction.

## 5. Centromere Inactivation, Reactivation and De Novo Centromere Formation

The chromosome type breakage-fusion-bridge (B-F-B) cycle was initiated by the fusion of two terminal deletion chromosomes, and resulted in a dicentric chromosome, which formed a bridge during anaphase that in turn would break to repeat the cycle. Translocation chromosomes between A and B chromosomes can undergo continuous centromere misdivision, nondisjunction, and chromosome type B-F-B cycles during cell divisions, resulting in a large number of centromere variant lines. A high frequency of centromere inactivation, generating stable maize dicentric chromosomes, was reported [31]. To produce the dicentrices, the TB-9Sb-Dp9 chromosome was constructed by recombining a duplicated chromosome 9 to the B–9 chromosome [32]. Intrachromosomal recombination can occur in the duplication of the 9S chromosome. Dicentric chromosomes with two B-repeat regions and one acentric fragment were observed during meiosis II. Only the chromosomes with one active and one inactive centromere could be stably transmitted to the next generation (Figure 2b). An inactive B centromere translocated to the tip of the short arm of chromosome 9 remained in the inactive state, without CENH3 labeling, over several generations [31]. The same DNA sequence in the dicentromeres provides additional evidence that DNA sequence is not the determinant for centromere function.

From a different perspective, a large-small dicentric chromosome was produced by crossing the TB-9Sb-Dp9 to T3-5 (+) with a truncated B centromere from a TB-9Sb misdivision line [33]. The large and small centromeres were in an active state. The foldback structure allowed recombination between the two chromosomes during meiosis and generated structures with two large centromeres and two small centromeres joined together. When the two centromeres were joined together in a stable chromosome that was inherited, the smaller centromere was inactive.

When this chromosome with a large active centromere and a small inactive centromere was studied, the foldback nature allowed the large and small centromeres to be separated from each other by recombination. They could be separated in meiosis I, and the two small-inactive centromere structures could be detected in the progeny, indicating that the centromere was reactivated at the original inactive centromere (Figure 2c). The CENH3 protein was indeed detected binding with one of the formerly inactive centromere regions. The reactivation process of the inactive centromere suggests that the centromere DNA-repeat sequences display a preference for kinetochore assembly, or other CENH3 chaperone factors such as the human holiday junction recognition protein (HJURP) in humans, the chromosome alignment defect 1 protein (CAL1) in *Drosophila*, or the suppressor of chromosome missegregation protein 3 (SCM3) in yeast [34].

A different process can be adopted for the transmission of this chromosome with two small inactive centromeres. De novo centromere formation in an ectopic region can regain centromere function instead of centromere activation, as has been reported in many other systems [35,36,37]. sDic15 is a dicentric chromosome that is generated from the process mentioned above, which includes intrachromosomal recombination and centromere reactivation (Figure 2 (d1)) [38]. No detectable CentC signals, and strongly reduced CRM and B-repeat signals, were observed in the centromere of the sDic15 mini-chromosome [38]. A 723 kb genomic region from chromosome 9 confers neocentromere function [38]. The misdivision of the chromosomes created breaks in the B centromeric region and produced many new centromere variants. The B centromere sequences were deleted but some B-repeat copies were still remaining in the 3–3 derivative. De novo centromere formation was detected in the 3–3 derivative with a 288 kb genomic region from chromosome 9 (Figure 2 (d2)). In a subsequent misdivision derivative of 3–3, a new centromere variant, without the previously formed neocentromere sequences, was detected; and other genomic regions, including an additional 200 kb of DNA sequences in chromosome 9, became the de novo centromere in derivative 3–3–11 (Figure 2 (d3)). These two neocentromeres were both misdivision derivatives of TB-9Sb, but with different centromere sequences. Centromere breakage and the B-F-B cycle process can both generate de novo centromeres.

The behavior of the maize B centromere, which includes centromere inactivation, reactivation and de novo centromere formation, reveals an epigenetic mechanism for centromere specification [39]. However, the molecular basis for these processes is still largely unknown. We have checked the DNA methylation state of the active and inactive B centromeres, and found that distinct DNA methylation patterns, between them, occur with a hypermethylation state in the inactive centromere [40]. The epigenetic difference is more likely to result after the formation of a stable chromosome state. Distinct DNA methylation patterns were also discovered in the satellite DNA sequences, between the pericentromere and centromere, in maize and *Arabidopsis thaliana* [41]. The comparison of DNA methylation in the ectopic region before and after de novo centromere formation showed a similar methylation level in this region, compared to normal centromeres, whether the original methylation level was high or low [38,42]. This indicates that there was maintenance of the centromere chromatin by an epigenetic mechanism. However, further work is needed on the detailed mechanism that establishes the centromeric chromatin, and on how centromere function is regained, as a de novo centromere or as a reactivation, at the original inactivation sites. Recently, the transcription and/or the transcripts from centromeric regions have been reported in many model systems, including maize [43,44,45,46,47]. They are involved in CENH3 loading, kinetochore assembly, and cell-cycle progression [48,49,50]. It may provide another aspect of the molecular basis of the transmission of different centromere chromatin states.

## 6. Meiotic Behavior of the Maize B Derivative Chromosome

Meiosis is a special cell division process that reduces chromosome numbers by half in the daughter cells. The centromere has an important role in meiosis I, homologous chromosome pairing, and segregation [51,52]. The orientation of sister chromatids is a key point for the process, especially the mono-orientation of the sister chromatids toward the same spindle pole in meiosis I [53]. The misdivision derivatives of TB-9Sb produce a series of B centromere deletion lines [54]. They provide a good model to study the meiotic behavior of centromeres with different sizes. Iwata-Otsubo et al. found that the CENP-A chromatin expansion, induced by the amplified centromere repeats, promoted increased transmission through the female germline in a model using mice [55]. We recently reported the observation of the combination of chromosomes with different sized B centromeres, during meiosis, in maize [56]. Only slightly higher frequencies, of a few percent, were observed for the larger centromere in several cases. However, the size of the centromeric DNA was not necessarily correlated with the signals of phH2AThr133 [56], suggesting the regulation of the functional size of the centromere.

**Figure 3:**
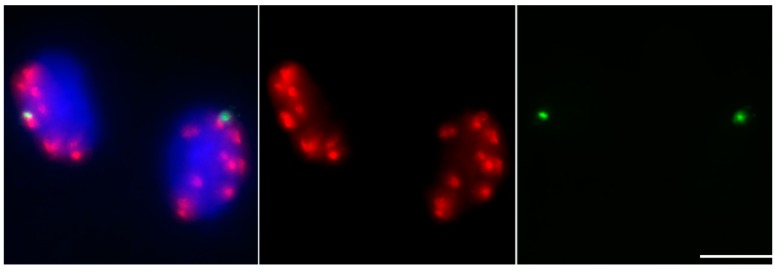
Labeling of phH2AThr133 in maize B mini-chromosome #9. The distribution of phH2AThr133 signals at telophase I stage of meiosis for maize B mini-chromosome #9. DAPI-stained chromosomes are blue, phH2AThr133 are red, and B-repeats are green. Bar = 10 μm. The B-repeat signals distributed in two cells in telophase I indicate that the sister chromatids of this mini-chromosome previously separated in anaphase I.

A number of mini-chromosomes were derived from the chromosome type B-F-B cycle of the TB-9Sb-Dp9 chromosome [31]. They ranged in both chromosome size and in the orientation of the sister chromatids during meiosis I [57]. The mini-chromosome derivatives 3–3 and sDic15 were generated from the maize B chromosome, through misdivision or intrachromosomal recombination, and de novo centromere formation was reported in these mini-chromosomes [38,58]. The mini-chromosomes #11 and sDic15 displayed mono-orientation in anaphase I, as the normal A chromosomes behaved mono-orientation in this stage. However, the mini-chromosome #3, #9, and derivative 3–3 showed bi-orientation, and their sister chromatids separated in anaphase I [38,57,58]. The distribution of B-repeat signals in two cells in telophase I indicated that the sister chromatids of the mini-chromosome #9 previously separated in anaphase I (Figure 3). What the differences are between the centromeres of the normal A chromosome and the different types of mini-chromosomes, and how they are distinguished for the molecular machinery of chromosome orientation, remain interesting questions.

All these mini-chromosomes contain functional centromeres with CENH3, phH2AThr133, phH3T3, and phH3Ser10 loading in mitotic cells [38,57,58,59]. The spindle assembly checkpoint kinase, ZmBub1, was detected in the mini-chromosome centromeres as normal A centromeres [60]. We speculate that the phosphorylation of histone H2AThr133 occurs during meiosis for these mini-chromosomes. The signal of phH2AThr133 loaded well in the mini-chromosome #9 (Figure 3), but no differences in phosphorylation levels were found in anaphase I during meiosis between the mini-chromosome #9, with mono-orientation or bi-orientation, and the normal A chromosomes [60]. The mechanical basis of the equational division of these mini-chromosomes may provide clues for the mechanism of chromosome orientation at meiosis I.

## 7. The Control of the Maize B Centromere Size

The canonical centromere size in any one species seems to assume a certain size, which is strongly correlated with genome size rather than the chromosome size [61]. The mean centromere size in rice is about several hundred kilobases, while the size of the maize centromere is about several megabases [8,62]. The centromere size is determined by the equilibrium between bound and unbound CENH3 proteins, which is regulated by genetic and epigenetic factors [63]. The maize centromere sizes expand to adjust to the oat background, in which the oat genome size is about 4-fold larger than the maize genome size [64]. The transcription of genes, and the corresponding epigenetic modifications, may restrict the centromere size [64,65].

The genome size will certainly be changed as one intact B chromosome is about the size of maize chromosome 10. However, accumulation of B chromosome does not change cell size as does ploidy. The maize B chromosome has been used to produce many lines with different sized centromeres. The CENH3-bound B centromeres may be not with expansion in the maize background even with high copy numbers of B chromosome. The smallest centromere size, resulting from misdivision, was only several hundred kilobase [66]. The small sized centromeres in maize B mini-chromosomes can be used to study the minimal DNA component required to assemble kinetochore. In addition, de novo centromeres from the misdivision lines were detected with centromeres ranging in size, and up to several hundred kilobases [58]. The centromeres of the mini-chromosomes are much smaller than the canonical maize centromere. However, the maize B centromere may expand when the B chromosome is introduced to the oat background as the oat-maize additional line. 

## 8. Concluding Remarks and Future Perspectives

The establishment and maintenance of the centromere chromatin is an interesting topic in chromosome biology. The molecular underpinnings of the transitions among the different chromatin states of the maize B chromosome centromere, including activation, inactivation and de novo centromere formation, is of great interest and is a good model to study. These processes may be involved in the coordination of complex epigenetic machineries that include DNA methylation, histone modification, chaperones, chromatin remodeling, and noncoding RNAs. Further work with the B centromere on these aspects will be carried out with the aid of maize B chromosome genome sequencing and the fruitful B centromere variants.
